# On the Factors Driving Upper-Ocean Salinity Variability at the Western Edge of the Eastern Pacific Fresh Pool

**DOI:** 10.5670/oceanog.2019.209

**Published:** 2019-06-14

**Authors:** J. Thomas Farrar, Albert J. Plueddemann

**Affiliations:** Department of Physical Oceanography, Woods Hole Oceanographic Institution, Woods Hole, MA, USA.

## Abstract

The tropical Eastern Pacific Fresh Pool (EPFP) has some of the highest precipitation rates and lowest sea surface salinities found in the open ocean. In addition, the sea surface salinity in the EPFP exhibits one of the strongest annual cycles in the world ocean. The region is strongly affected by the meridionally migrating Intertropical Convergence Zone and is also influenced by large-scale ocean currents and wind-driven Ekman currents. Recognizing the complexity of competing regional influences and the importance of sea surface salinity as an integrator of freshwater forcing, the Salinity Processes Upper-ocean Regional Study (SPURS) was undertaken to better understand how ocean processes and surface freshwater fluxes set surface salinity. Instrumentation on a surface mooring, deployed for 14 months near the western edge of the EPFP, allowed estimation of the surface fluxes of momentum, heat, and freshwater. Subsurface instrumentation on the mooring provided upper-ocean vertical structure and horizontal currents. These observations, along with horizontal gradients of surface salinity from the Soil Moisture Active Passive (SMAP) satellite instrument, were used to estimate the surface-layer salinity budget at the western edge of the EPFP. While the low salinity associated with the presence of the EPFP at the mooring site was sustained by heavy rainfall, it was found that seasonal variability in large-scale currents was important to controlling the transition between the “salty” and “fresh” seasons. Ekman advection was important to prolonging local high salinity as rainfall decreased. Although illuminating some key processes, the temporal variability of the surface-layer salinity budget also shows significant complexity, with processes such as surface freshwater fluxes and vertical mixing making notable contributions. The surface flux term and the terms involving mixing across the base of the surface layer oppose and nearly cancel each other throughout the deployment, such that the horizontal advection term effectively accounts for most of the variability in surface salinity at the site on monthly to seasonal timescales. Further investigation, taking advantage of additional observations during SPURS-2, will be needed to more thoroughly examine the relevant physical processes.

## INTRODUCTION

**FIGURE 1.**
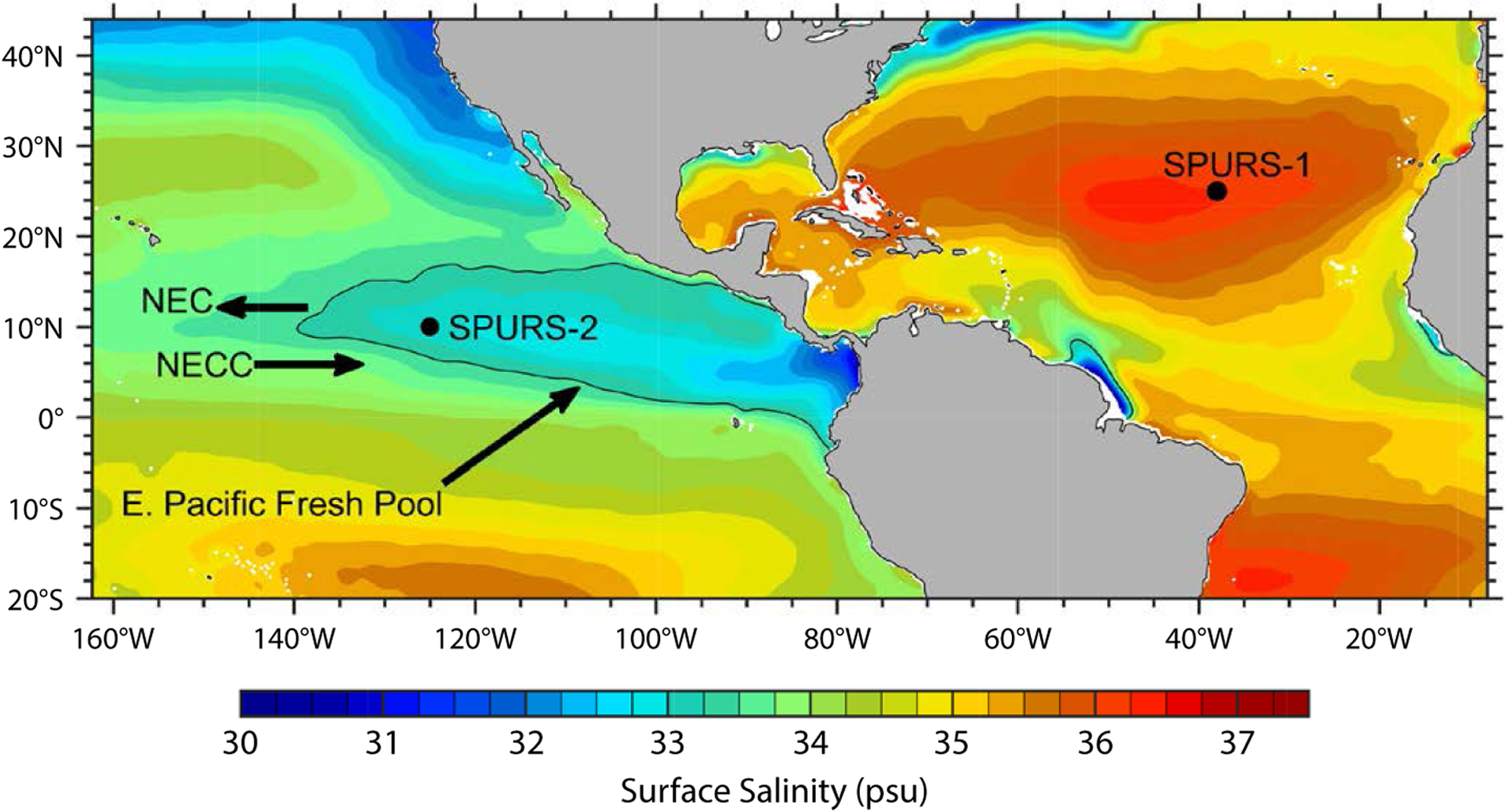
The 2016–2017 mean surface salinity from the Soil Moisture Active Passive (SMAP) satellite instrument. The sites of the SPURS-1 and SPURS-2 air-sea interaction moorings are marked by black circles. The SPURS-2 central mooring site is in the western part of the climatological Eastern Pacific Fresh Pool (EPFP; defined here as the 34 psu isohaline, shown as a black contour), but the EPFP expands and contracts seasonally and interannually under the influence of rainfall, currents, and other factors. The zonally oriented North Equatorial Current (NEC) and North Equatorial Counter Current (NECC), which influence the seasonal evolution of the EPFP, are indicated schematically with arrows. *Figure after* Guimbard et al. (2017)

The majority of global evaporation and precipitation takes place over the ocean. The sea surface salinity field reflects the integrated effect of this evaporation and precipitation, but it is also affected by oceanic transports of salt. The fact that the ocean integrates the freshwater forcing makes ocean salinity a potentially powerful indicator of changes in the global water cycle (e.g., Durack and Wijffels, 2010; Terray et al., 2012). Motivated by the similarities and differences between patterns of surface freshwater fluxes and surface salinity, the Salinity Processes Upper-ocean Regional Study (SPURS) was undertaken to better understand how ocean processes and surface freshwater fluxes set surface salinity. Two field campaigns were conducted, SPURS-1 in the salinity maximum region of the subtropical North Atlantic (Lindstrom et al., 2015), and SPURS-2 in the salinity minimum zone of the eastern tropical Pacific Ocean (Figure 1).

**FIGURE 2.**
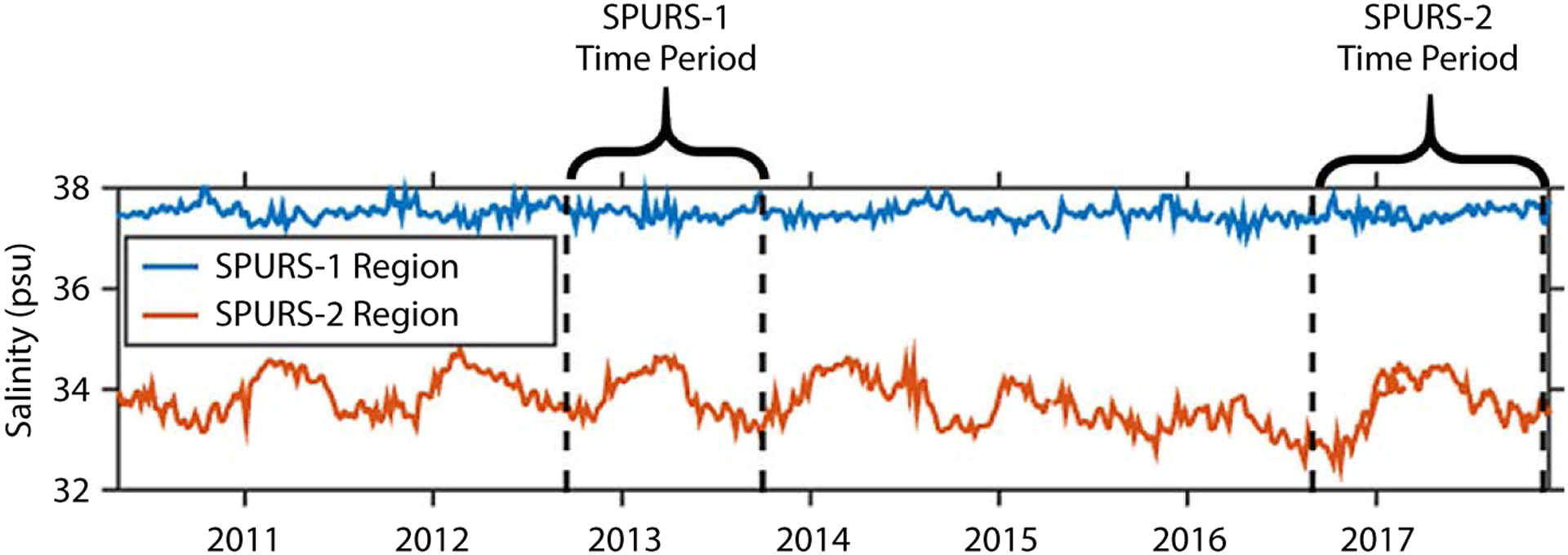
Sea surface salinity (SSS) at the SPURS-1 and SPURS-2 sites (blue and orange curves, respectively). The salinity data are from the Soil Moisture and Ocean Salinity (SMOS) satellite (until early 2017; CNES-IFREMER product; Reul et al., 2015) and the SMAP satellite (after April 2016; Meissner et al., 2018).

The SPURS-1 and SPURS-2 experiments were deliberately conducted in regions exhibiting strong contrasts in surface salinity, surface freshwater forcing, and ocean dynamics. The SPURS-1 site was in the North Atlantic subtropical gyre, a region of high salinity and relatively weak spatial gradients. The variability of sea surface salinity (SSS) through the year at the SPURS-1 site is minimal. The SPURS-2 site, on the western edge of the tropical Eastern Pacific Fresh Pool (EPFP; Alory et al., 2012), is characterized by some of the highest precipitation rates and the lowest sea surface salinities found in the open ocean; it also has one of the strongest annual cycles of SSS in the world ocean. The contrast in SSS evolution at the two sites is clear from inspection of time series of SSS (Figure 2). The SPURS-1 site exhibits an exceedingly weak annual cycle in SSS (Sena Martins et al., 2015) that is not even discernible in some years (Figure 2, lower panel). In contrast, the SPURS-2 site exhibits a very strong annual cycle in SSS, with an annual range typically exceeding 1 psu.

In this paper, we present a first look at the mooring measurements from SPURS-2 and a preliminary diagnosis of how the balance of surface fluxes, horizontal advection, and mixing changed during the yearlong mooring deployment. The first section presents more detail on hypotheses about the role of precipitation and horizontal advection in the Eastern Pacific Fresh Pool that helped to motivate the experimental design of SPURS-2. It is followed by a description of the data used, a brief description of the surface meteorology and surface salinity as seen in the mooring record, and the approach we used to estimate the relative influence of different factors on the surface-layer salinity. We then present the results of the surface-layer salinity balance and discuss the results.

## THE EASTERN PACIFIC FRESH POOL AND IMPORTANT HYPOTHESES OF THE SPURS-2 PROGRAM

The tropical Eastern Pacific Fresh Pool (Alory et al., 2012; Guimbard et al., 2017) is a major focal point of the SPURS-2 program. The EPFP can be defined as the region of the eastern tropical Pacific where SSS is less than 34 psu (Guimbard et al., 2017). We deployed a surface mooring on the western edge of the EPFP (at 10°N, 125°W; Figure 1) to determine the air-sea fluxes of freshwater, heat, and momentum. This region lies beneath the summertime location of the atmospheric eastern Pacific Intertropical Convergence Zone (ITCZ) and has the highest average rainfall rates in the open-ocean eastern Pacific. Analysis of precipitation data from the Global Precipitation Climatology Project (Adler et al., 2003) showed that there is approximately 3 m of rainfall per year at the site (SPURS-2 Planning Group, 2015).

Several competing influences on surface salinity may be important at the mooring site, and the balance between them is expected to change seasonally. The eastern Pacific Intertropical Convergence Zone migrates north and south between about 5°N (Northern Hemisphere winter) and 12°N (Northern Hemisphere summer), and brings its heaviest precipitation at 10°N from August to October, which would tend to decrease salinity. At the same time (August–October), the wind stress curl field of the ITCZ promotes Ekman upwelling while the ITCZ is over-head, which would bring salty subsurface waters closer to the surface where they can be mixed into the surface layer. When the ITCZ is to the south of the mooring site during February to April, the northeast trade winds are strong, causing enhanced evaporation and enhanced northward Ekman transport, both of which would tend to increase salinity. The westward-flowing North Equatorial Current (NEC) and eastward-flowing North Equatorial Counter Current (NECC) are also potentially major influences. The 10°N site is in between the climatological average locations of the NECC (5°–8°N) and NEC (10°–15°N), but the two currents move north and south and vary in intensity seasonally with the ITCZ and trade winds (e.g., Farrar and Weller, 2006; Hasson et al., in press).

Recent studies enabled by satellite salinity measurements have stimulated hypotheses about how precipitation, evaporation, and ocean dynamics work together to drive variability in SSS at different times and locations in and around the EPFP. One of our goals in SPURS-2 is to test and improve upon these previous hypotheses with field observations collected during SPURS-2. Prior to SPURS-2, we posed the important hypothesis that northward Ekman transport associated with the westward trade winds persistently drives a northward transport of the freshwater that is deposited under the Northern Hemisphere ITCZ (Yu, 2015; Tchilibou et al., 2015). Thus, when the ITCZ is moving northward in spring, the low SSS region and the ITCZ precipitation move northward together. But, when the ITCZ precipitation is moving southward in the fall, the low SSS water continues to move northward, despite the fact that the ITCZ and its persistent high precipitation rates are moving southward (Yu, 2015). This hypothesis is based partly on a zonally averaged analysis, a perspective that neglects zonal gradients and zonal advection, which may not be appropriate in the northeast tropical Pacific where there is an appreciable zonal gradient associated with the EPFP as well as intense, seasonally variable zonal currents associated with the NEC and NECC. Guimbard et al. (2017) present an alternative to the zonal-averaged view—they propose that the seasonal cycles of the NEC and NECC are controlling factors in the seasonal cycle of SSS in the SPURS-2 region.

## DATA AND METHODS

### Air-Sea Interaction Mooring Data

A Woods Hole Oceanographic Institution (WHOI) surface mooring was deployed at 10°N, 125°W to provide high-quality measurements of surface meteorology for making accurate estimates of the surface fluxes of heat, momentum, and freshwater. Beneath the surface, the mooring made densely spaced, high-frequency measurements of subsurface temperature, salinity, and velocity. The mooring and buoy instrumentation setup was very similar to that used in SPURS-1 (Farrar et al., 2015). The mooring was deployed for more than 14 months (August 2016–November 2017) and recorded two rainy seasons when the ITCZ was present at the site (June–November), a period when the northeast trade winds were present and the ITCZ was south of the mooring (January–June), and the local departure and arrival of the EPFP as it expanded and contracted seasonally.

The buoy carried two independent Improved METeorological (IMET) systems (Hosom et al., 1995; Colbo and Weller, 2009; Bigorre et al., 2013). Each IMET system consists of a central data logger connected to autonomous sensor modules measuring downward solar radiation (Eppley Standard Precision Pyranometer), downward infrared radiation (Eppley Precision Infrared Radiometer), air temperature and humidity (Rotronic MP-101A), barometric pressure (Heise DXD), wind speed and direction (R.M. Young 5103 Wind Monitor), precipitation (R.M. Young 50202 Self-siphoning Rain Gauge), and sea surface temperature and salinity (Sea-Bird SBE-37). Accuracies for each module and the associated accuracy implied for air-sea flux estimates are given by Colbo and Weller (2009). In addition to the two IMET systems, we also outfitted the buoy with additional backup instruments for key variables (rain, air temperature, and humidity). The buoy also carried a WHOI low-power direct-covariance flux system (DCFS) and a directional wave package for estimating surface-wave directional spectra.

After recovery of the mooring, we carefully evaluated the redundant records and constructed a best-estimate time series. The meteorological and sea surface variables were used with a bulk flux algorithm (COARE 3.0; Fairall et al., 2003) to estimate the air-sea fluxes of heat, momentum, and moisture.

There were more than 60 subsurface instruments on the mooring line for measuring temperature, salinity, and velocity. All of the subsurface temperature and salinity instruments sampled at an interval of five minutes or less. The vertical spacing of temperature-salinity instruments was about 3 m in the upper 25 m and became progressively coarser with depth (increasing to about 5 m over depths of 25–90 m and reaching about 20 m at depths 110–160 m). There were several current profilers and current meters on the mooring. Of interest for this study were a 300 kHz RDI Workhorse acoustic Doppler current profiler (ADCP) deployed looking upward from 75 m depth to measure the vertical profile of horizontal currents over depths spanning about 9 m to 70 m with 2 m vertical bins, and a Nortek Aquadopp point current meter measuring currents at 3 m depth. Both profilers had a sampling interval of 30 minutes. These two records were combined and were linearly interpolated in depth to estimate the vertical profile of horizontal velocity over the upper 70 m.

The overall data return was excellent, but there were some data quality issues, especially related to biofouling of temperature-conductivity (salinity) sensors in the upper 5 m late in the 14-month deployment. A preliminary correction was applied to minimize apparent salinity drift in some sensors near the end of the record. The results shown here are insensitive to the details of the correction, or even to whether it is used at all. This insensitivity might seem surprising, given that the horizontal and vertical salinity gradient in the upper 5 m can be substantial in the rainy and low-wind conditions found in the region (Boutin et al., 2016). Three factors explain this insensitivity of the monthly timescale salinity budget to the sensor accuracy in the upper 5 m: (1) the conditions that give strong salinity stratification in the upper few meters are transient (e.g., rain events), and we are focusing on salinity variations on much longer monthly timescales, (2) the sensor drift (about 0.08 psu/month) is not large compared to the upper-ocean salinity signals of interest (~0.3 psu/month), and (3) our analysis focuses on the salinity integrated over the mixed layer (>50 m), so the salinity errors in the upper 5 m make a relatively small contribution to the layer-averaged salinities.

### Observed Evolution of Surface Forcing and Surface Salinity

**FIGURE 3.**
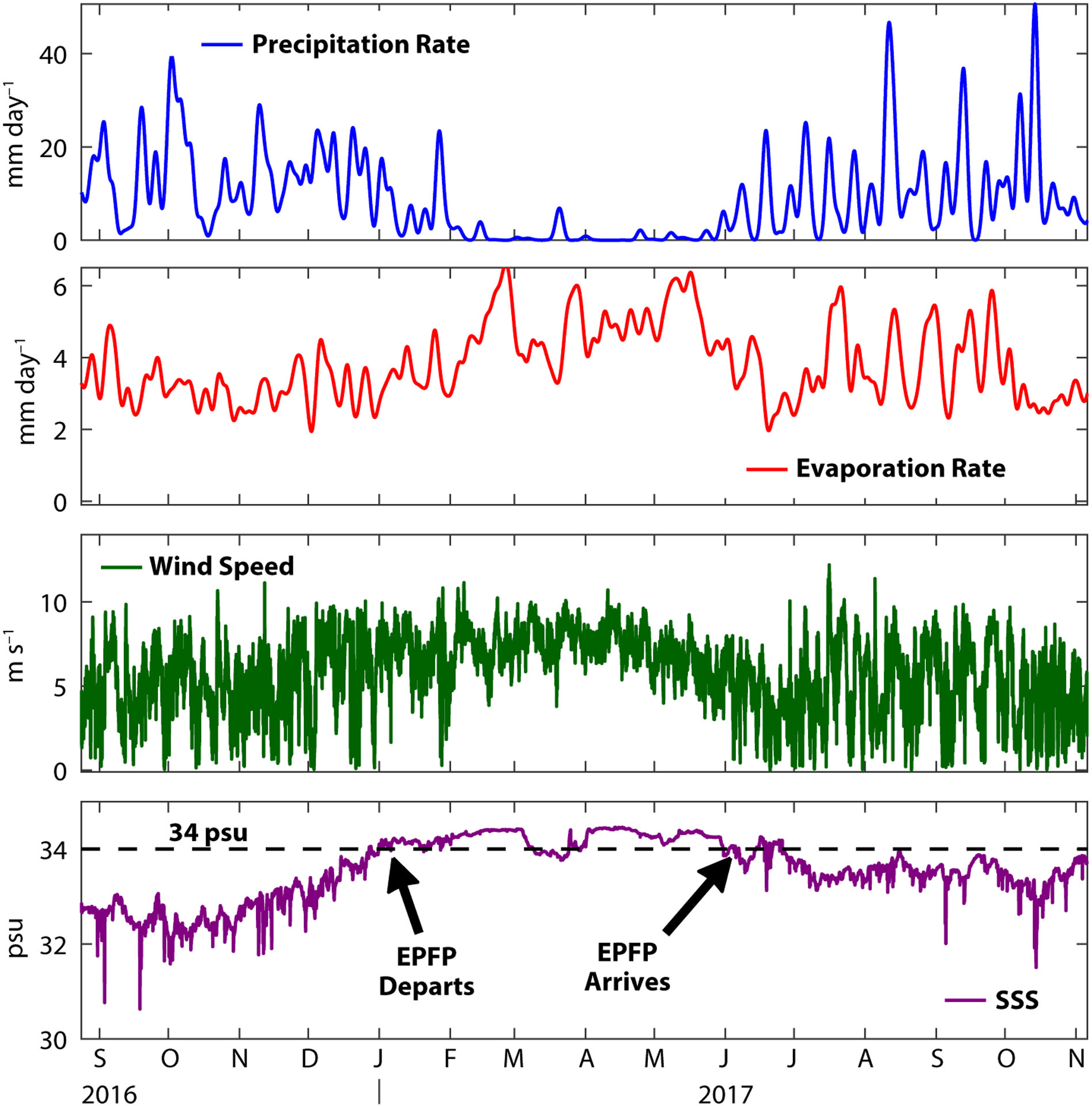
Measurements and derived quantities from the SPURS-2 air-sea interaction buoy (from top to bottom: five-day average rain rate, five-day average evaporation rate, wind speed, and SSS). The 14-month deployment recorded two rainy seasons when the Intertropical Convergence Zone (ITCZ) was present at the site (June–November), a period when the northeast trade winds were present and the ITCZ was south of the mooring (January–June), and the local departure and arrival of the Eastern Pacific Fresh Pool (marked in lower panel; the EPFP is present when SSS is below 34 psu).

The mooring was deployed in August 2016 during the rainy season. Surface salinities were low (around 33 psu), and the mooring site was solidly within the EPFP. Wind speed was variable and frequent, heavy rainfall events continued until around January 2017, and five-day average rainfall rates frequently exceeded 10 mm/day (Figure 3). Around January 1, 2017, the wind speed became more steady and the precipitation began to taper off, signaling that the ITCZ had moved south of the mooring site and been replaced by the northeast trade winds. From February to May 2017, the winds were remarkably steady, and the evaporation rate was high. In June 2017, the ITCZ returned, with its precipitation and variable winds.

The measured surface salinity appears to reflect the precipitation rate, in the sense that there were low salinities when it was raining (Figure 3). This is not exactly what would be expected for the local SSS response to seasonal rainfall, in the sense that the lowest salinities would be expected to occur at the end of the rainy season (when the accumulated precipitation plateaus). In fact, close comparison of the changes in salinity to the rainfall rate reveals that, near the end of the 2016 rainy season, the surface salinity started increasing while the rain rate was still high and the evaporation rate was low. Moreover, at the beginning of the 2017 rainy season, the SSS started decreasing before there had been very much rainfall. The surface-layer salinity balance discussed below can help us understand these apparent contradictions.

During the first 12 months of the deployment, the total amount of rainfall was about 3.0 m, very high by global standards and consistent with expectations for a mooring location beneath the eastern Pacific ITCZ (SPURS-2 Planning Group, 2015). Interestingly, the total evaporation was also remarkably large, about 1.4 m over the first year. For comparison, in the evaporation-dominated SPURS-1 region in the subtropical Atlantic, the total evaporation over the first year was 1.6 m (Farrar et al., 2015). Most of the evaporation at the SPURS-2 site occurred during February–June, when the strong, steady trade winds were present.

The same mooring site was occupied by another WHOI surface mooring about a decade earlier (1997–1998), and analyses of the earlier record provide some additional context. The site exhibits strong eddy variability with an annual cycle tied to the instability of the NEC (Farrar and Weller, 2006). During the ITCZ season, with its variable winds, there can be strong diurnal variations in upper-ocean temperature (Prytherch et al., 2013) and energetic episodes of mixed-layer near-inertial oscillations (Plueddemann and Farrar, 2006).

### Surface Layer Salinity Budget at the SPURS-2 Mooring

The local surface layer salinity budget at the SPURS-2 mooring site can provide important insights into the regional dynamics at the westward edge of the EPFP. We will use the SPURS-2 data together with satellite data to estimate the terms in the surface layer salinity balance, and we will use the surface layer salinity budget to help identify important physical processes influencing upper ocean salinity in the SPURS-2 region.

The surface layer salinity budget can be written as (e.g., Foltz et al., 2004)

(1) $$\frac{\partial S}{\partial t}=-\vec{u}\cdot \nabla S+{\hat{S}}_{-h}\left(\frac{\partial h}{\partial t}\right)-\frac{Q_{-h}}{h}\;+\frac{E-(P+C)}{h}S_{o}+R,$$

where *S* is defined as the layer-average salinity, $\vec{u}$ is the layer-average horizontal velocity, quantities with hats indicate the vertically-varying deviation from the average, the subscript *−h* denotes the value at the depth *h*, *Q*_*−h*_ is the vertical turbulent salinity flux at depth *h*, *E* is evaporation from the sea surface, *P* is precipitation, *C* is condensation on the sea surface, and *S*_*o*_ is the surface salinity. The residual, *R*, represents terms that should be included in the equation but that can-not be readily estimated from available data. The terms on the right-hand side represent, from left to right, (1) horizontal advection of the layer-average salinity by the layer-average current, (2) part of the vertical entrainment term (further discussed below), (3) changes in salinity driven by the vertical turbulent heat flux of salt across the layer base, (4) the surface forcing term, with changes in layer-average salinity driven by evaporation, condensation, and precipitation at the sea surface, and (5) the residual term (discussed below). The depth *h* could be any arbitrary depth; for a focus on upper-ocean salinity, it is sensible to choose *h* to be the mixed layer depth or the depth of an isopycnal in the upper pycnocline. For this preliminary investigation, we define the surface layer depth as the depth where the potential density increases by 0.4 kg m^−3^ relative to the surface (Figure 5).

To estimate the horizontal gradients of surface layer salinity, we used data from the NASA Soil Moisture Active Passive (SMAP) mission. The specific data product used was the eight-day running mean, 40 km gridded product produced by Remote Sensing Systems (Meissner et al., 2018). The basic assumption under-lying this choice is that the vertical gradient of salinity is small enough within the surface layer that the surface salinity is a good proxy for the vertically averaged salinity within the layer.

**FIGURE 4.**
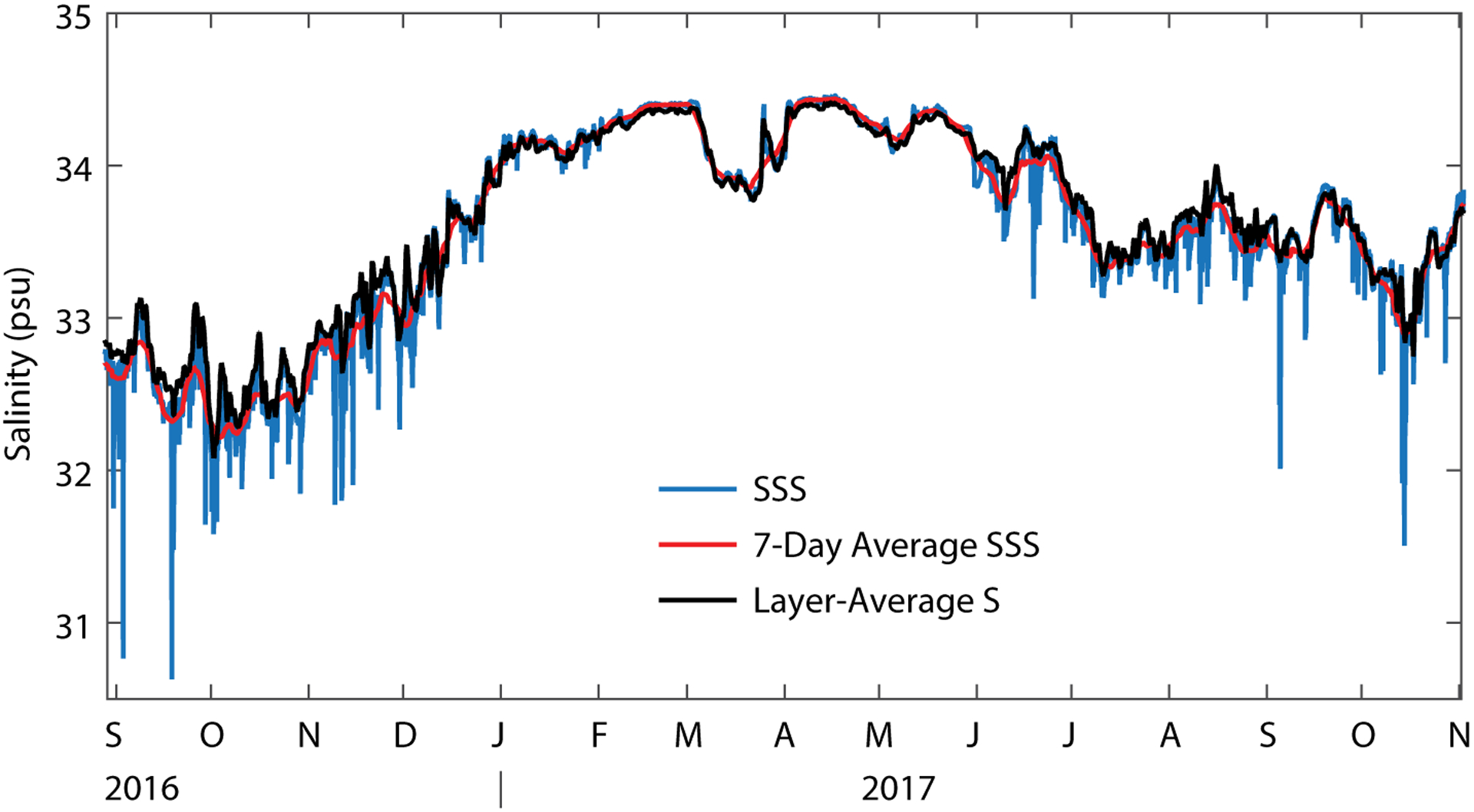
Comparison of different representations of surface salinity from the SPURS-2 air-sea interaction buoy: hourly-average surface salinity (blue line), seven-day average surface salinity (red line), and the layer-average salinity (defined as the vertical average of salinity over the “surface layer” that extends to the depth where potential density increases by 0.4 kg m^−3^ relative to the surface value; black line).

**FIGURE 5.**
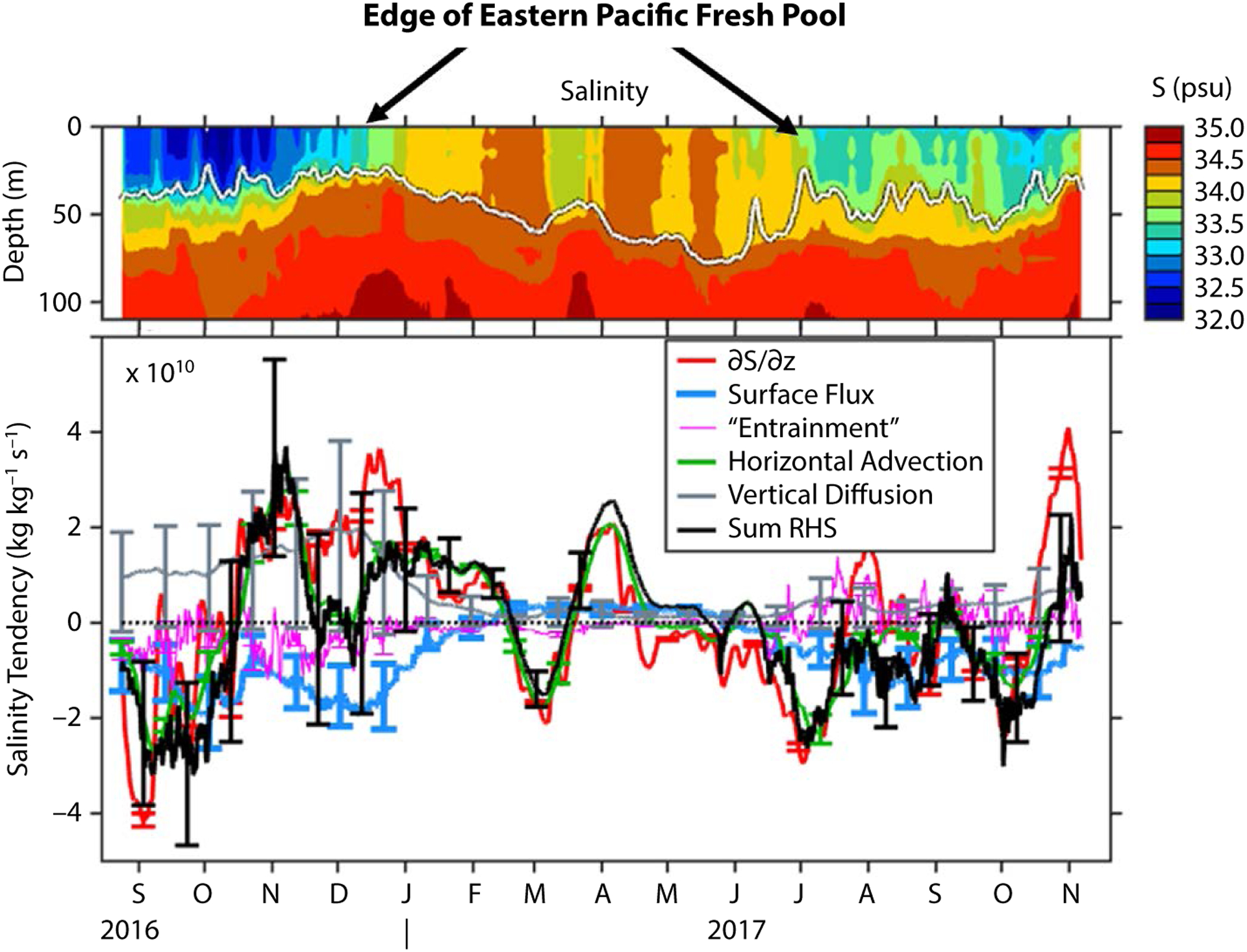
(upper panel) Subsurface salinity measured at the mooring site, as a function of depth and time. The layer depth, *h*, that was used for the salinity budget (white line) is the depth where the potential density increases by 0.4 kg m^−3^ relative to the surface value. (lower panel) Preliminary evaluation of the surface layer salinity budget at the SPURS-2 air-sea interaction mooring site. The local departure and arrival of the Eastern Pacific Fresh Pool (salinity <34 psu) is marked in the upper panel. The black line, denoted “sum RHS” in the figure legend, is the sum of the terms related to surface fluxes, horizontal advection, “vertical entrainment,” and vertical diffusion at the base of the layer (i.e., the sum of all terms except the residual term, *R*, on the right-hand side of Equation 1). The units in the bottom panel are 10^10^ kg of salt per kg of seawater per second—one of these units is roughly 0.26 psu/month.

The validity of the assumption that surface salinity can serve as a proxy for layer-average salinity in the horizontal gradient estimate deserves further discussion. Comparison of the layer-average salinity to the seven-day running mean salinity (red and black lines in Figure 4) shows that the two curves are in fact difficult to distinguish from one another. This close similarity between the weekly averaged surface salinity and the layer-average (vertically averaged) salinity might seem surprising because of the attention given in SPURS-2 to shallow fresh layers deposited on the surface during rain events (e.g., Drushka et al., 2019, in this issue). However, one can indeed see a dramatic difference caused by sporadic, short-duration rain events when comparing the layer-average salinity to the hourly surface salinity values from the buoy (Figure 4); the rain events are visible as brief downward spikes in the surface salinity. We can interpret the close similarity of weekly averaged surface salinity and layer-average salinity as an indication that on the weekly and longer timescales of interest here, the surface layer as we have defined it is relatively well mixed and weakly stratified (see also Figure 5, lower panel), with the intermittent presence of fresh lenses having a minor impact. In this context, the horizontal gradients of surface salinity provide an acceptable approximation to the horizontal gradients of layer-average salinity, but this is of course dependent on the particular definition of “surface layer” used here.

Using hourly average data from the mooring and the SMAP gridded surface salinity described above, we estimated the rate of change of surface salinity (left-hand side of Equation 1) and the horizontal advection, partial vertical entrainment, and evaporation-condensation-precipitation terms on the right-hand side of Equation 1. We also made a crude estimate of the vertical turbulent flux of salt across the layer base, modeling it as a diffusive flux (*Q*_*−h*_ = κ *∂S*/*∂z*) with a vertical eddy diffusivity, κ, of 2 × 10^−5^ m^2^ s^−1^. The particular value used is within the large range of values previously reported from microstructure in the eastern equatorial Pacific (summarized by Inoue et al., 2012). A more careful treatment of this term will be an important avenue for future work in SPURS-2 analysis. Finally, we applied a 31-day running average to the result.

We estimated the errors in each of these terms following Farrar et al. (2015). Briefly, we estimated the standard error for each term by propagating the estimated instrumental errors through Equation 1, accounting for both random and bias errors and the 31-day averaging. The main difference from the error estimates in Farrar et al. (2015) is for the term representing the turbulent flux at the layer base; to bound the errors for this term, we varied the assumed turbulent diffusivity from 4 × 10^−7^ m^2^ s^−1^ to 4 × 10^−5^ m^2^ s^−1^.

The residual term, *R*, is the difference between the rate of change of layer-average salinity and the sum of computed terms on the right-hand side. In addition to the accumulation of estimation errors, the residual contains two terms not estimated explicitly here:

(2) $$R={\hat{S}}_{-h}\left(w_{-h}+\vec{u_{-h}}\cdot \nabla h\right)\;-\frac{1}{h}\nabla \cdot \int_{-h}^{0}\hat{S}\vec{\hat{u}}dz.$$

These terms are difficult to estimate because they involve horizontal gradients of subsurface quantities, which are difficult to resolve observationally. The first term on the right-hand side of *R* is the part of the entrainment term involving vertical and horizontal advection of the surface-layer base. The complete vertical entrainment term (the sum of the second term on the right-hand side of Equation 1 and the first term on the right-hand side of Equation 2 accounts for water that enters or leaves the layer across the layer base (e.g., because of upwelling or turbulence-driven mixed-layer deepening). Because we are only estimating part of the entrainment term in Equation 1, we will refer to that term with quotes (i.e., as the “vertical entrainment” term) because the estimated term captures only part of that process. The second term in the residual is sometimes referred to as the stratified-shear term, and it tends to be small when the layer depth, *h*, is chosen so that there is only weak salinity stratification within the layer (as in this case). The neglected terms, along with errors in the estimated terms, will be reflected in the mismatch between the estimated rate of change of layer average salinity (*∂S*/*∂t*) and the sum of the other estimated terms.

## RESULTS

During the first two months of the deployment (mid-August to mid-October, 2016), the rate of change of surface layer salinity (Figure 5) was large and negative (about −2 × 10^−10^ kg kg^−1^ s^−1^, which is about 0.52 psu/month), meaning that the surface layer was rapidly freshening. Around mid-October of 2016, the surface layer began to rapidly become more salty (at a rate of about +2 × 10^−10^ kg kg^−1^ s^−1^), and this persisted until mid-February. There was then a brief, one-month period of freshening that peaked around March 1, after which the salinification trend resumed and continued until around May. From July until October 2017, the surface layer was mostly getting fresher, although the rate of change of salinity was quite variable, with periods of both salinification and freshening. During the last month of the record, the salinity again began to increase rapidly.

Evaluation of the terms in the surface layer salinity balance (Figure 5) allows us to understand how evaporation, precipitation, vertical mixing, and lateral transport of fresher or saltier water by ocean currents affected the evolution of upper-ocean salinity. The rate of change of surface layer salinity is nearly balanced by the sum of the terms related to surface fluxes, horizontal advection, “vertical entrainment,” and vertical diffusion at the base of the layer, indicating that these factors can largely explain the observed changes in salinity. The surface flux term, the horizontal advection term, and the vertical diffusion term all play important roles, while the “vertical entrainment” term is less important. The residual term, visualized by the difference between the red and black lines in Figure 5, is occasionally large (e.g., November–December 2016 and October–November 2017), indicating that some important processes are not captured in our budget, either because of estimation errors or because of contributions from the terms that were not explicitly estimated.

The surface forcing term acts on average to freshen the surface layer salinity at the site, as expected for one of the rainiest places in the world. Note that surface layer depth, *h*, appears in the denominator of the surface flux term in Equation 1, which means that surface fluxes are more effective in changing the salinity when the layer is thin. For example, the effect of the heavy rainfall during the rainy season when the ITCZ is over the mooring site (June–December) is amplified by the shallow mixed layer depths that occur during this time period.

In addition to the 3.0 m of precipitation that fell during the first year of the mooring deployment, there was also a significant amount of evaporation (about 1.4 m). Evaporation was particularly strong during the trade wind season (February–May) because of strong, steady winds and low humidity. However, because the mixed layer was relatively deep at this time, the excess evaporation only weakly influenced the surface layer salinity (Figure 5). (The effect of condensation on the sea surface was small in comparison to evaporation and precipitation and is not discussed here.)

The term involving the vertical turbulent flux at the layer base, modeled here as a turbulent diffusion, was also an important contributor to the salinity balance. The vertical diffusion term was of comparable strength to the surface flux term throughout the study period, and it was especially important during September–December 2016. During this period, the mixed-layer salinity was low and the salinity gradient at the mixed-layer base was strong—the strong vertical gradient contributed to a larger flux of salt into the surface layer that largely counteracted the local freshwater input from rainfall. In addition, the turbulent flux term was more effective in changing the surface-layer salinity during this time (September–December 2016) because of the relatively shallow layer depth.

Interestingly, the surface flux term and the vertical mixing terms (vertical diffusion and entrainment) very nearly balance one another throughout the deployment. As a consequence, the temporal variations in the rate of change of layer-average salinity closely resemble the variations in the horizontal advection term. Although several terms are large, the horizontal advection term appears to account for most of the variability in surface salinity at the site on the timescales considered here (monthly to seasonal).

## DISCUSSION

Knowing which terms make important contributions to the evolution of surface layer salinity is only a start toward our ultimate goal of understanding the different physical processes and phenomena that affect upper-ocean salinity. The surface layer salinity budget indicates which processes are important at different times (horizontal advection, surface fluxes, vertical mixing), and we would like to then understand which phenomena are causing these processes. For example, is the horizontal advection signal driven by the strong equatorial zonal currents or by the persistent northward Ekman currents? A more detailed look at the SPURS-2 observations can help to answer these questions.

The increase of salinity above 34 psu in November–December 2016 heralded the eastward retreat of the EPFP. One intriguing feature of the SPURS-2 mooring record is that this local increase of salinity occurred despite heavy rainfall. Diagnosis of the surface layer salinity balance suggests that this occurred because subsurface turbulent fluxes and advection were acting to increase the surface layer salinity, and these influences combined to overwhelm the influence of the heavy rainfall in the ITCZ. Advection also appears to have played an important role in the re-appearance of the EPFP at the site in June 2017: the decrease of salinity below 34 psu in June 2017 was driven initially by advection of fresh water from elsewhere, followed by local rain.

**FIGURE 6.**
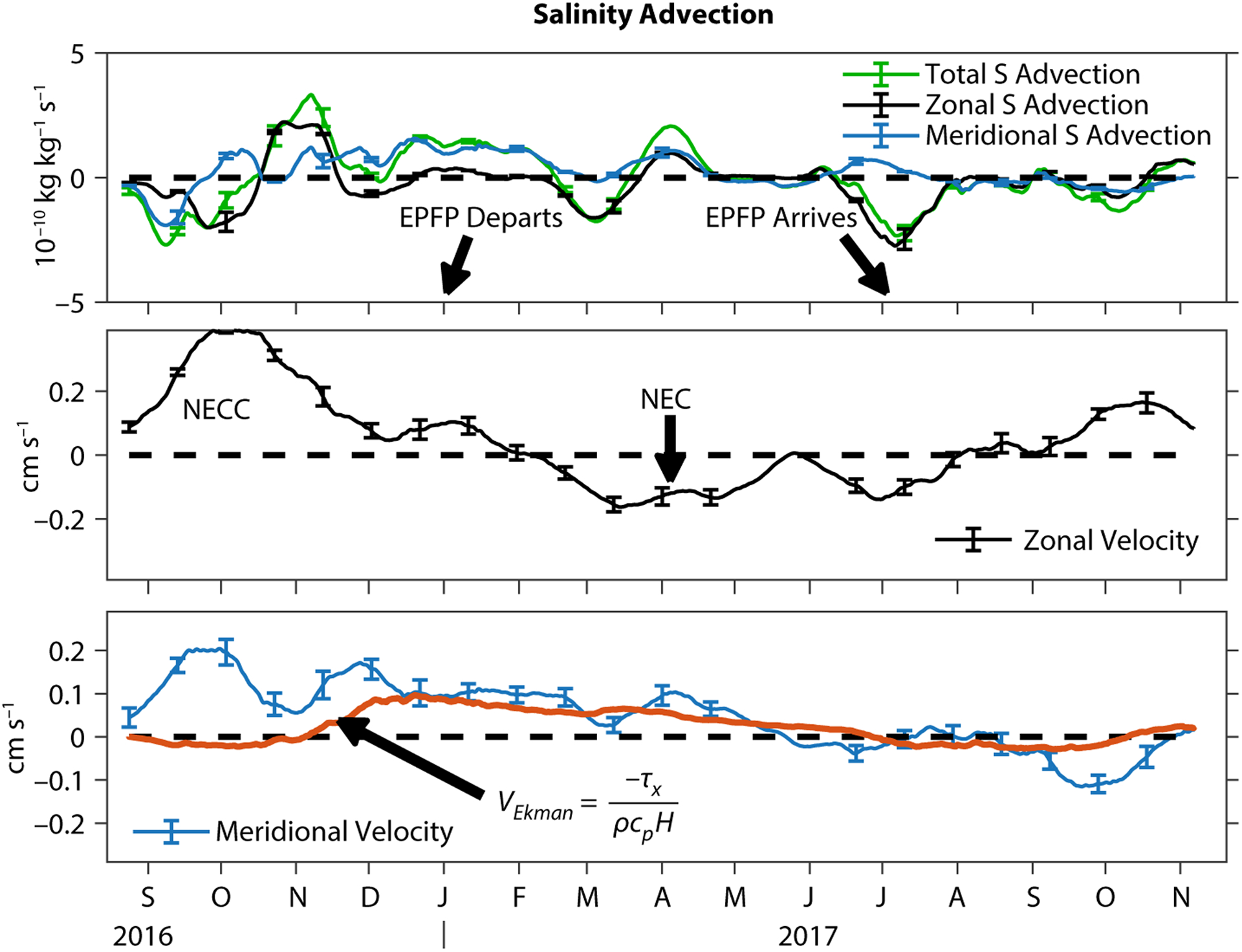
(top panel) Decomposition of the salinity advection term into zonal and meridional contributions (the advection term, defined in Equation 1, describes the layer-averaged currents acting on satellite-derived horizontal salinity gradients). Times of the local departure and arrival of the Eastern Pacific Fresh Pool (EPFP) are indicated by arrows. (middle panel) Layer-averaged zonal velocity. (lower panel) Layer-averaged meridional velocity (blue line), with an estimate of the layer-averaged northward Ekman current derived from the surface wind stress (red line).

Two of the causes of horizontal advection that have been hypothesized to be important in the annual evolution of the location of the EPFP are northward Ekman advection (Yu, 2015) and zonal advection by the NEC and NECC (Guimbard et al., 2017). The mooring data can be used to decompose the salinity advection term into zonal and meridional components to allow more detailed insights into the local influence of northward Ekman advection and the strong zonal equatorial currents (Figure 6). The total salinity advection term is quite variable in time, with several reversals from positive to negative values.

The influence of zonal advection was variable and was only intermittently strong (Figure 6, upper panel). One of the times when it was strong, though, coincided with the local arrival of the EPFP in July 2017. At this time, advection was acting to reduce the local surface layer salinity (Figure 6, upper panel), and the zonal currents were relatively strong and flowing to the west (Figure 6, middle panel). Thus, the zonal currents were carrying fresh water from the east, contributing to the westward expansion of the EPFP and its arrival at the mooring site in July 2017. The SPURS-2 salinity budget from the mooring indicates that it was only later, in August 2017, that the heavy local rainfall began to contribute significantly to low-ering the salinity.

At the time when the EPFP disappeared from the mooring site, in January 2017, meridional advection was acting to increase the salinity locally, and zonal advection was weak, consistent with the Ekman advection hypothesis (Figure 6, top panel). The meridional currents responsible for this meridional advection were northward and remained relatively steady from January to May 2017, as would be expected for Ekman transport driven by the westward trade winds. A more quantitative test of the idea that the observed northward flow is due to wind-driven Ekman transport can be made by comparing the layer-averaged northward current to the theoretical Ekman transport for the layer (Figure 6, lower panel). Assuming that the turbulent momentum flux across the base of the surface layer is small, the vertically integrated Ekman current is expected to be *V*_*Ek*_ = −*τ*_*x*_/(*ρfh*), where *f* is the local value of the Coriolis parameter, *ρ* is the water density, and *τ*_*x*_ is the zonal component of the wind stress. The theoretical meridional Ekman current is a reasonably good match to the observed layer-averaged meridional current during the trade wind season of January–May (Figure 6, lower panel). During the January–June period, the fluctuations of the observed meridional velocity around the theoretical Ekman current at periods of one to two months are likely associated with the intraseasonal eddies that are prevalent in this region (Farrar and Weller, 2006; Hasson et al., in press). The agreement of the theoretical northward Ekman current and the observed meridional velocity is notably worse during September–November 2016 and from September 2017 to the end of the record—it is not clear why this happens, but the explanation could involve intraseasonal eddies or some other dynamics associated with the NEC/NECC current system.

While these results support the hypothesis that northward Ekman transport contributes to the local disappearance of the EPFP and the continued salinification during the trade wind season, the salinity balance estimated from the SPURS-2 mooring data paints a more complicated picture. Local freshwater fluxes and vertical mixing were exerting as strong an influence as meridional advection at the time when the EPFP was leaving the SPURS-2 mooring site (Figure 5).

To summarize, the surface-layer salinity budget estimated from the SPURS-2 central mooring supports the following conclusions:

- SPURS-2 was in a highly dynamic region, with strong SSS variability, precipitation, evaporation, and currents.
- The transition between salty and fresh seasons coincided with the local NEC/NECC seasonal cycle (similar to Guimbard et al, 2017), but the low SSS in the EPFP was sustained locally by heavy rainfall.
- Ekman advection contributed pro-longed salinification after the ITCZ moved to the south (similar to Yu, 2015).
- Vertical turbulent flux appears to be important (as large as local surface flux), particularly during the anomalously fresh fall of 2016.
- The surface flux term and the vertical mixing terms (vertical diffusion and entrainment terms) nearly cancel one another throughout the deployment, such that the horizontal advection term effectively accounts for most of the variability in surface salinity at the site on monthly to seasonal timescales.

There is reasonably good closure of the surface-layer salinity budget from the terms estimated here, which can provide some insights into the influences on surface salinity in the region, but the most interesting times might be those when the salinity budget is not balanced. The residual appears to be largest during the time when the edge of the EPFP is either arriving at or retreating from the site (November–December 2016 or July–August 2017). During these transitional times, there are stronger horizontal gradients of salinity and a more complicated (less uniform) vertical structure in the upper ocean, both of which are favorable conditions for contributions from the “stratified-shear term” (Equation 2) that was not explicitly estimated here. Horizontal fluxes of salt by submeso-scale instabilities and horizontal inter-leaving of different water masses would be reflected in the stratified-shear term. Understanding the contributions of these processes to the salinity and heat balances will be an important avenue for future work in SPURS-2 and other studies.

We would like to extend the analysis just presented to examine the physical processes in more detail and to more fully utilize other SPURS-2 observations. For example, the gliders deployed by University of Washington researchers carried temperature microstructure instruments that can provide more direct estimates of the contribution from turbulent mixing, which was only crudely parameterized in the foregoing analysis. Similarly, it will be interesting to compare the buoy flux measurements discussed here to the direct measurements of turbulent surface fluxes in SPURS-2 (Clayson et al., 2019, in this issue) Another way to complement the Eulerian view afforded by the moorings, the satellites, and some of the autonomous assets would be by more extensive analysis of the “Lagrangian drift” experiment carried out during SPURS-2 (Lindstrom et al., 2017) that used mobile autonomous platforms (gliders and Wave Gliders) and drifters to follow a Lagrangian float being carried eastward into the EPFP by the NECC.
